# Alpha 1-antitrypsin mitigates salt-sensitive hypertension in juvenile mice by reducing diacylglycerol concentrations and protein kinase C activity in kidney membranes

**DOI:** 10.3389/fmolb.2024.1485506

**Published:** 2025-01-20

**Authors:** Yunus E. Dogan, Niharika Bala, Erika S. Galban, Russell L. Lewis, Nancy D. Denslow, Sihong Song, Abdel A. Alli

**Affiliations:** ^1^ Department of Medicine Division of Nephrology, Hypertension, and Renal Transplantation, University of Florida College of Medicine, Gainesville, FL, United States; ^2^ Department of Physiology and Aging, University of Florida College of Medicine, Gainesville, FL, United States; ^3^ Department of Pediatrics, Faculty of Medicine, Erciyes University, Kayseri, Türkiye; ^4^ Department of Physiological Sciences and Center for Environmental and Human Toxicology, University of Florida College of Veterinary Medicine, Gainesville, FL, United States; ^5^ Department of Pharmaceutics, University of Florida College of Pharmacy, Gainesville, FL, United States

**Keywords:** protein kinase C, diacylglycerols, alpha-1 antitrypsin, salt-sensitive hypertension, sodium-potassium-chloride co-transporter

## Abstract

**Introduction:**

Recombinant alpha-1 antitrypsin (AAT) therapy has been shown to have beneficial effects to mitigate the progression of various diseases. Here, we hypothesized that administration of pharmaceutical-grade human AAT (hAAT) is effective in mitigating hypertension induced by salt-loading in juvenile mice by reducing the concentration of diacylglycerols (DAGs) and activity of protein kinase C (PKC) in the kidney.

**Methods:**

Four-week old 129Sv mice were salt-loaded to induce hypertension and then administered hAAT or vehicle.

**Results:**

Administration of hAAT was found to significantly reduce high blood pressure in both the active and inactive cycles of the 129Sv hypertensive mice. A lipidomic analysis showed decreased concentrations of multiple diacylglycerols in kidney cortex membrane fractions from mice treated with hAAT compared to vehicle. PKC activity was less in the 129Sv mice that received hAAT compared to vehicle. Western blotting and immunohistochemistry analysis showed the density of the sodium-potassium-chloride co-transporter (NKCC2) was significantly reduced in kidney cortex membrane fractions of juvenile mice that received hAAT compared to vehicle.

**Conclusion:**

Taken together, this study demonstrates a new protective effect of hAAT in normalizing blood pressure after the development of saltinduced hypertension in juvenile mice in a mechanism involving a decrease in NKCC2 membrane expression, presumably due to decreased levels of DAGs in the plasma membrane and a subsequent decrease in PKC activity.

## 1 Introduction

Hypertension in children is an increasingly recognized public health concern, with an estimated prevalence ranging from 2% to 5% in the pediatric population, and elevated blood pressure affecting approximately 13%–18% of children and adolescents globally (Yang et al., 2021). Recent systematic reviews indicate that the pooled prevalence of hypertension among children and adolescents is about 4.00%, with notable increases observed over the past two decades, primarily associated with rising rates of obesity (Song et al., 2019). The causes of juvenile hypertension can be categorized into primary (essential) and secondary hypertension. Primary hypertension is more common in older children and is often linked to genetic factors and lifestyle choices, such as obesity, physical inactivity, and dietary habits. In contrast, secondary hypertension can arise from identifiable medical conditions, including renal disease or endocrine disorders, but it is less prevalent, accounting for about 10%–14% of cases in the pediatric population (Hsu et al., 2021). The development of juvenile hypertension is recognized to be a potential precursor of hypertension in adulthood (Meng et al., 2024). Early identification and successful treatment of juvenile hypertension may prevent the development of multiple organ damage (Hinton et al., 2020). Childhood-onset hypertension is linked to subclinical cardiovascular disease and persists throughout adulthood (Myette and Flynn, 2024; Robinson et al., 2024). Children with hypertension have an increased risk of stroke, hospitalization for myocardial infarction or unstable angina, coronary intervention, and congestive heart failure. The risk of adult cardiovascular disease may be decreased by managing childhood hypertension (Robinson et al., 2024).

Alpha-1 antitrypsin (AAT) is a multifunctional protein that is known to have anti-inflammatory effects and protective effects in various organ systems and tissues. AAT administration has been shown to prevent type 1 diabetes in a non-obese diabetic mouse model (Song et al., 2004), reduce cigarette smoke-induced emphysema in mice (Pemberton et al., 2006), lower blood pressure in diabetic mice (Lugo et al., 2022), and delay the development of arthritis in mice (Grimstein et al., 2011). The role of AAT in mitigating diabetic kidney disease and hypertension secondary to diabetes has not been thoroughly studied.

Several blood pressure regulating proteins have been shown to be affected by protein kinases C (PKC) activity. Free radical superoxide has been shown to stimulate PKC and increase apical plasma membrane expression of the Na^+^-K^+^-2Cl^−^ cotransporter (NKCC2) (Haque and Ortiz, 2019). PKC-mediated phosphorylation was shown to regulate the activity and function of the with-no-lysine kinase 4 (WNK4), which in turn phosphorylates the kinase Ste20-related proline alanine-rich kinase (SPAK) and oxidative stress-responsive kinase (OSR1), leading to the phosphorylation and activation of the renal Na-Cl cotransporter (NCC) (Castaneda-Bueno et al., 2017). Multiple studies have shown PKC negatively regulates renal epithelial sodium channels (ENaC). Bao et al. showed ENaC activity is elevated in native split-open cortical collecting ducts of PKC alpha knockout mice (Bao et al., 2014). Other studies have shown PKC negatively regulates ENaC activity and its density at the luminal membrane by phosphorylating the myristoylated alanine-rich C kinase substrate (MARCKS)/MARCKS like protein 1 (MLP1) protein, causing its translocation to the cytoplasm (Alli et al., 2012; Montgomery et al., 2017; Song et al., 2020).

Diacylglycerols (DAGs) are bioactive lipids that are known to activate specific isoforms of PKC. The availability of DAG to function as a second messenger depends on its regulation by other proteins. For example, diacylglycerol kinase (DGK) terminates DAG signaling by converting DAG to phosphatidic acid, which by itself is involved in signal transduction (Cai et al., 2009).

Here we investigated for the first time the efficacy of administering human AAT (hAAT) to mitigate salt-induced hypertension in juvenile non-obese 129Sv mice and investigated whether a potential mechanism for this protective effect of hAAT could be due to the suppression of PKC activity as a result of decreased levels of the bioactive lipid DAG. Mechanistically, we investigated whether a decrease in DAG and PKC is associated with a decrease in surface expression of renal NKCC2 to presumably mitigate sodium retention and alleviate salt-induced hypertension.

## 2 Materials and methods

### 2.1 Experimental design

**FIGURE 1:**
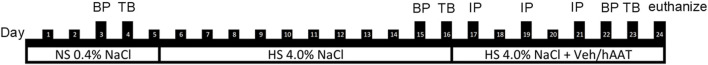
Schematic of study design. Each vertical line represents 1 day. Blood pressure (BP), tail bleeds (TB), intraperitoneal (IP) injection of hAAT or vehicle. There were 3 phases: normal salt (NS), high-salt (HS), and high-salt plus treatment with hAAT or vehicle.

Juvenile mice were initially maintained on a normal-salt diet for 5 days and then switched and maintained on a high-salt diet for 11 days. Hypertension from the salt-loading was confirmed by tail-cuff blood pressure measurements, and then after the 11th day the mice were given either vehicle (VEH) or hAAT via intraperitoneal injection while the mice were maintained on a high salt diet. The mice were euthanized on day 24 of the study, and the kidneys were harvested for protein biochemistry experiments (Figure 1).

### 2.2 Animals

Male (N = 9) 129Sv mice were purchased from the Jackson Laboratory (Bar Harbor, ME, United States). The mice were 4 weeks old at the start of the study. All our animal studies were performed under an approved University of Florida Institutional Animal Care and Use Committee Protocol Number 202011157 and these studies were in compliance with the National Institutes of Health “Guide for the Care and Use of Laboratory Animals”.

### 2.3 Blood pressure measurements

Since blood pressure is known to be regulated in a time-of-day dependent manner (Alli et al., 2019), blood pressure measurements were performed using the tail-cuff method (IITC MRBP System from Life Science Inc.; Woodland Hills, CA, United States) in the AM (6 a.m.–8 a.m.) during the animals inactive cycle and in the PM (6 p.m.–8 p.m.) during the animals active cycle. Blood pressure was taken on the third day of the normal salt diet of 0.40% NaCl (Teklad, Envigo, Indianapolis, IN, United States) (Figure 1). To induce hypertension in juvenile 129Sv mice, the mice were fed a high-salt diet of 4.0% NaCl (Teklad, Envigo) for 11 days. Blood pressure was measured on the 10th day of the high salt diet (Figure 1). The mice were maintained on the high-salt diet and were given hAAT by intraperitoneal (IP) injection every other day. The blood pressure was taken after the fifth day of the treatment phase with hAAT or vehicle by intraperitoneal (IP) injection.

### 2.4 Tail vein blood collections

The lateral tail vein was cannulated with a 22 g needle and microhematocrit tubes were used to collect the blood during the active and inactive cycles. The blood was subjected to centrifugation at 6,000 × g RCF for 5 min and the plasma was collected and stored at −20°C.

### 2.5 Drug treatment

Salt-induced hypertensive 129Sv mice were given either vehicle or clinical grade hAAT (Prolastin®C, Grifols, hAAT (2 mg/mouse/every other day) or vehicle (0.9% sterile saline (Fisher Scientific) via intraperitoneal (IP) injection. The vehicle group consisted of 4 male 129Sv mice while the hAAT treated group consisted of 5 male 129Sv mice. The mice received one injection at 9 a.m., every other day, for a total of three injections.

### 2.6 Tissue processing

The right kidney was divided into 50-mg slices and cut longitudinally. Each segment was then homogenized in 500 µL of tissue protein extraction reagent (TPER) (Thermo Fisher Scientific) using an Omni TH homogenizer. The lysates were subjected to a 20-minute incubation on ice and were vortexed every 5 min. Following incubation, the tissue lysates were subjected to centrifugation at a speed of 15,800 × g RCF for a duration of 10 min using a Micromax benchtop centrifuge (Thermo IEC). The liquid portion was collected and underwent ultracentrifugation using an Optima L-90K ultracentrifuge (Beckman Coulter; Schaumburg, IL, United States) at a speed of 109,600 × g RCF for 30 min at a temperature of 4°C. This was done using a SW55.1 rotor (Beckman Coulter). The supernatant was collected and constituted the soluble fraction. Afterwards, 250 µL of TPER was utilized to dissolve the pellet, which was then subjected to sonication for two 5-second intervals. This process resulted in the formation of the membrane fraction. The protein concentration of the soluble and membrane fractions was determined using a BCA assay (Thermo Fisher Scientific).

### 2.7 Western blotting

Fifty micrograms of total protein were applied to 4%–20% gradient SDS PAGE gels (ThermoFisher Scientific) to analyze the soluble fractions or membrane fractions. The proteins were separated by electrophoresis for a duration of 1 h using a Criterion apparatus (BioRad). The proteins were deposited onto nitrocellulose membranes using a Criterion apparatus (BioRad). The membranes were blocked in a solution containing 5% milk and 1X Tris-Buffered Saline (1X TBS) for 1 h to reducing nonspecific binding. Afterwards, the membranes were incubated with a 1:1,000 dilution of primary anti-NKCC2 antibody (18970-1AP) (Proteintech; Rosemont, IL) prepared in a solution containing 5% bovine serum albumin (BSA) as the carrier protein and 1X TBS, and the incubation was done overnight at a temperature of 4°C. The membranes underwent 3 washes with 1X TBS before being placed in a solution of secondary antibody (1:3000 dilution in blocking solution). The blots were incubated with enhanced chemiluminescence (ECL) reagent (BioRad) for a duration of 5 min, after which the blots were imaged (BioRad imager).

### 2.8 Lipidomics

Lipids were extracted from kidney cortex membrane preparations as described in Section 2.6 by the Bligh & Dyer method (Bligh and Dyer, 1959) with the following modifications. Samples were quantitated using 1 internal standard for each lipid class. The concentration of each lipid was reported as ng/mL. Lipidomics was performed as previously described by our group (Gholam et al., 2023) with the following modifications. The EquiSPLASH Lipidomix (Avanti Polar Lipids, Inc., Alabaster, AL, United States) mixture was used as an internal standard, which consists of a mix of 13 deuterated lipids, each at 100 μg/mL concentration. Membrane preparations were taken and placed into the −80°C freezer until ready for extraction. Samples were pre-normalized to 100 µg protein, aliquoting between 7 and 13 µL sample volume per sample on ice. A phenomonex Luna NH2 100 mm x 2 mm column, 3 µm particle size. Part # 00D-4377-B0 was utilized and analysis was performed on an ABSCIEX 6500 QTRAP LC MS/MS instrument using scheduled MRM. A normal phase separation was carried out and acquired with Analyst 1.7 hotfix 3 software and quantitated using MultiQuant 3.0.3 software.

### 2.9 Protein kinase C activity assay

A protein kinase C activity assay (abcam) was performed using kidney cortex membrane fractions lysates while following the instructions from the manufacturer.

### 2.10 Immunohistochemistry

Kidney sections embedded in paraffin were first immersed in Xylene (ThermoFisher Scientific) twice for 3 min each. They were then subjected to a series of 3-minute treatments with ethanol at concentrations of 100%, 95%, 75%, and 50% (ThermoFisher Scientific). After a 3-minute rinse in autoclaved distilled water, the slides were incubated in boiling citrate buffer for 20 min. Following a 5-min rinse in 1X PBS (Phosphate Buffered Saline), tissue sections were blocked with 2.5% normal horse serum (Vector Laboratories) for 20 min at room temperature in a humidified chamber. The slides were then incubated with the primary antibody (NKCC2, Proteintech), diluted 1:500 in 1X PBS, for 30 min at room temperature in the humidified chamber. After a 3-minute rinse in 1X PBS, the slides were incubated with the VectorFluor Duet Reagent secondary antibody (Vector Laboratories Inc.) for 30 min in the dark. This was followed by three 3-minute rinses in 1X PBS. Finally, the slides were cover-slipped (Genesse Scientific) using Vectashield anti-fade mounting medium with DAPI (Vector Laboratories Inc.) and imaged with a fluorescence Olympus microscope using a 40× objective.

### 2.11 Statistical analysis

A Student t-test was performed to compare differences between the hAAT and vehicle groups. If the normality test failed a Mann-Whitney Rank Sum test was performed to determine statistical significance between the two groups. A one-way ANOVA was performed to compare the blood pressure data. The SigmaPlot software 15.0 (Systat Software, San Jose, CA, United States) was used for all statistical analyses. The values presented are expressed as mean ± SE, and a *p*-value of less than 0.05 was considered statistically significant.

## 3 Results

### 3.1 hAAT decreases systolic blood pressure in hypertensive juvenile 129Sv mice

**FIGURE 2:**
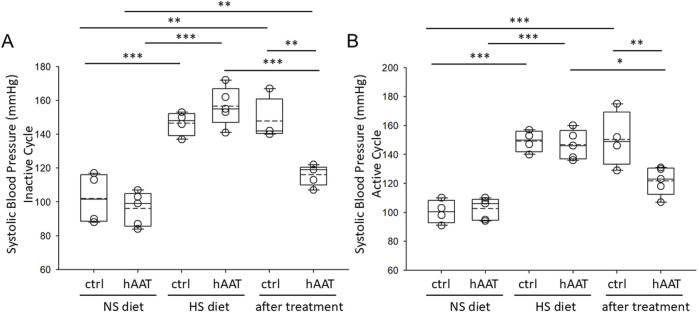
Systolic blood pressure measurements in juvenile 129Sv mice treated with vehicle or hAAT before and after salt-loading. **(A)** summary plot of the systolic blood pressure during the inactive cycle, **(B)** summary plot of the systolic blood pressure during the active cycle. A one-way ANOVA was used to compare differences between the groups. ***p < 0.001, **p < 0.01, *p < 0.05.

hAAT was previously shown to be efficacious in mitigating hypertension in diabetic db/db mice (Lugo et al., 2022). However, the efficacy of hAAT in alleviating salt-sensitive hypertension in juvenile mice has not been studied. After salt-loading for 10 days, 129Sv mice became hypertensive presumably from the retention of sodium followed by an increase in extracellular fluid volume. Here we show that hAAT significantly reduced salt-induced hypertension in both the active and inactive cycles of juvenile 129Sv mice (Figure 2).

### 3.2 hAAT treatment alters the concentration of lipids in kidney cortex membrane fractions

**FIGURE 3:**
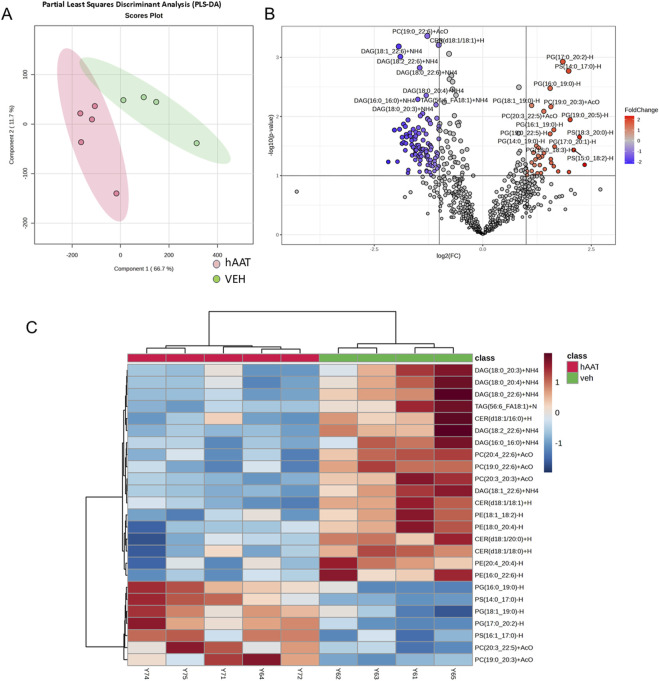
Lipidomic analysis of kidney cortex membrane fractions from 129Sv mice treated with vehicle or recombinant hAAT. Data normalized to median with Pareto data scaling. **(A)** Partial least squares discriminant analysis (PLS_DA) score plot. **(B)** Volcano plot showing lipids that are differentially enriched in 129Sv mice treated with vehicle (VEH) or hAAT. **(C)** Heatmap of the top 25 lipids that were differentially enriched between the two groups. N = 4 mice for the vehicle group and N = 5 mice for the hAAT group. MetaboAnalyst 6.0 software (Pang et al., 2024) was used to create the PLSDA score plot, volcano plot, and heatmap.

A previous study suggested that the administration of hAAT in adult diabetic mice alters the concentration of various lipids in the kidney (Lugo et al., 2022). Here we investigated for the first time whether hAAT administration affects the abundance of various lipids in the kidney of juvenile salt-induced hypertensive mice. A volcano plot and heatmap of the lipidomic data shows that the concentrations of several different classes of lipids including multiple bioactive lipids are altered in kidney cortex membrane fractions of 129Sv mice treated with hAAT compared to vehicle (Figure 3). Diacylglycerols represented the largest class of bioactive lipids that were decreased in availability in kidney cortex membrane fractions after hAAT treatment (Figure 3). Other bioactive lipids that were down-regulated included various ceramides.

### 3.3 The concentration of multiple forms of diacylglycerols is reduced in membrane fractions of the kidney cortex of hypertensive 129Sv mice after administration of hAAT compared to vehicle

**FIGURE 4:**
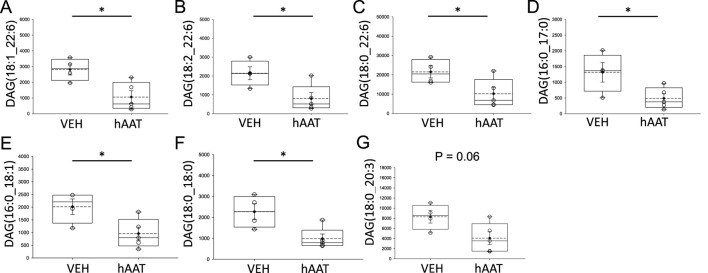
Analysis of diacylglycerol concentrations within kidney cortex membrane fractions from 129Sv mice treated with vehicle or recombinant hAAT. A student’s t-test was performed using the raw data values from the lipidomics dataset after normalization to the median. VEH represents vehicle. hAAT represents human alpha-1 antitrypsin. DAG forms that were significantly downregulated after hAAT administration included **(A)** DAG (18:1_22:6), **(B)** DAG (18:2_22:6), **(C)** DAG (18:0_22:6), **(D)** DAG (16:0_17:0), **(E)** DAG (16:0_18:1), **(F)** DAG (18:0_18:0), **(G)** DAG (18:0_20:3). A Student t-test was performed to compare differences between the two groups. N = 4 mice for the vehicle group and N = 5 mice for the hAAT group. * *p*-value <0.05.

Diacylglycerols occur in various forms within the plasma membrane of renal epithelial cells and play an important role as second messengers in physiology and pathophysiology. Here, our lipidomic analysis showed multiple forms of diacylglycerols, including DAG (18:1_22:6), DAG (18:2_22:6), DAG (18:0_22:6), and DAG (16:0_17:0), DAG (16:0_18:1), DAG (18:0_18:0), DAG (18:0_20:3) are all significantly decreased in concentration in kidney cortex membrane fractions of juvenile 129Sv mice treated with hAAT compared to mice with vehicle (Figure 4). Other lipids that were detected are listed in Supplementary Table S1.

### 3.4 PKC activity is significantly attenuated in kidney cortex membrane fractions of 129Sv mice treated with hAAT compared to vehicle

**FIGURE 5:**
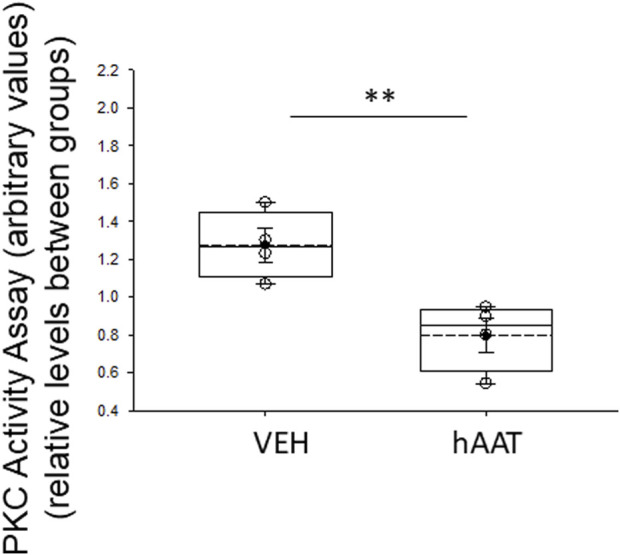
Protein kinase C activity in membrane fraction kidney cortex lysates of 129Sv mice treated with vehicle or recombinant hAAT. VEH represents vehicle. hAAT represents human alpha-1 antitrypsin. A Student t-test was performed to compare differences between the two groups. N = 4 mice for the vehicle group and N = 5 mice for the hAAT group. ** *p*-value<0.01.

Since our lipidomic data showed hAAT administration decreases the availability of multiple forms of DAGs, and DAGs are known to activate PKC isoforms, we investigated whether PKC activity in kidney cortex membrane fractions is also altered by hAAT treatment. As shown in Figure 5, PKC activity was significantly downregulated in membrane fractions of the kidney cortex from 129Sv mice treated with hAAT compared to vehicle.

### 3.5 hAAT administration reduces NKCC2 membrane expression in the kidney of juvenile 129Sv mice

**FIGURE 6:**
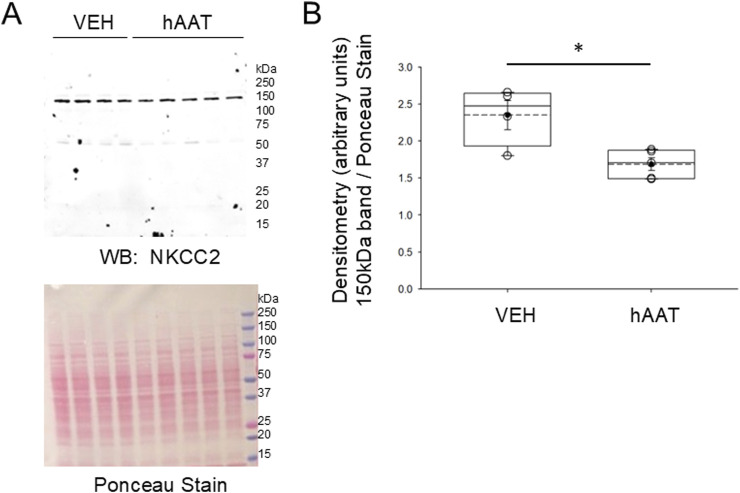
Western blot of NKCC2 protein expression in the kidney of 129Sv mice treated with hAAT or vehicle. **(A)** Representative Western blot (top) for NKCC2 in kidney cortex membrane fractions after salt-loaded hypertensive juvenile mice were treated with vehicle (VEH) or hAAT. N = 4 mice in the VEH group and N = 5 mice in the hAAT group. Ponceau stain (bottom) used to assess lane loading. **(B)** Densitometry plot of the immuno-reactive NKCC2 band normalized to the Ponceau stain. A Student’s t-test was performed to compare differences between the two groups. * represents a *p*-value <0.05.

**FIGURE 7:**
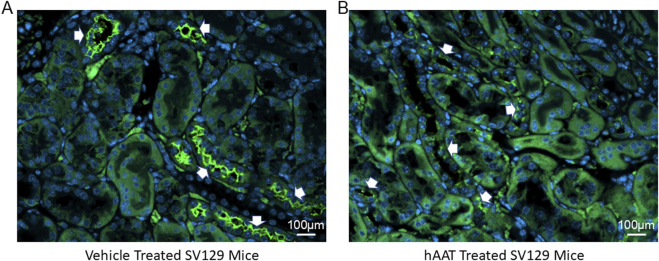
Immunohistochemistry of NKCC2 protein expression in the kidney 129Sv mice treated with vehicle or hAAT. **(A)** Representative images of vehicle treated salt-loaded hypertensive juvenile 129Sv mice. **(B)** Representative images of hAAT treated salt-loaded hypertensive juvenile 129Sv mice. N = 4 mice per group. White arrows indicates positive staining for NKCC2 in luminal/apical plasma membrane of the kidneys of each group of mice. Scale bar for all images is 100 µm.

Since the surface expression of NKCC2 is known to be enhanced by phosphorylation of the actin cytoskeleton protein annexin A2 (Dathe et al., 2014), which is also regulated by proteolysis (Yamane et al., 2013), we investigated for the first time whether of the versatile protease inhibitor AAT affects the density of NKCC2 protein expression in kidney cortex membrane fractions of juvenile salt-induced hypertensive mice. As shown in Figure 6, hAAT administration significantly reduced NKCC2 protein expression in kidney cortex membrane fractions of juvenile 129Sv mice compared to vehicle treatment. To corroborate the Western blot results showing a decrease in NKCC2 protein expression in kidney cortex lysates of 129Sv juvenile mice given hAAT, we performed immunohistochemistry using paraffin-embedded tissue. As shown in Figure 7, luminal plasma membrane staining of NKCC2 was significantly attenuated in the kidneys of salt-induced hypertensive juvenile 129Sv mice treated with hAAT compared to mice treated with vehicle.

## 4 Discussion

Our study demonstrates for the first time that hAAT treatment alters the concentrations of multiple classes of lipids in the membranes of kidneys from hypertensive juvenile 129Sv mice. A previous study from our group showed hAAT attenuated ENaC and MARCKS protein expression along with ceramide metabolism to hexosylceramides in renal epithelial cells to alleviate salt-induced hypertension in adult diabetic db/db mice (Lugo et al., 2022). However, this is the first study to investigate the efficacy and mechanism of hAAT administration to mitigate hypertension in juvenile mice.

In an attempt to address this knowledge gap, we designed a study to investigate changes in the concentration of membrane lipids from salt-induced hypertensive juvenile 129Sv mice treated with hAAT or vehicle. Our lipidomics data identified several bioactive lipids that were downregulated in the kidney cortex membrane fractions of hypertensive 129Sv mice that received hAAT compared to vehicle. Diacylglycerols represented the largest class of bioactive lipids that were downregulated in the kidney membranes after administration of hAAT. Importantly, diacylglycerols play an important role in the activation of specific PKC isoforms including PKC alpha, beta, delta, and epsilon.

It is known from previous studies that DAG serves as the primary stimulant of protein kinase C in biological processes (Ohanian and Ohanian, 2001), and acts as an allosteric activator of protein kinase C (PKC), while IP3 primarily regulates intracellular calcium levels by modulating calcium channels in both the plasma membrane and endoplasmic reticulum. This result is particularly important in relation to smooth muscle contraction (Berridge, 1987). At the same time Okumara et al. concluded that 1,2-diacylglycerol production by norepinephrine is elevated in the thoracic aorta of spontaneously hypertensive before the onset of hypertension (Okumura et al., 1990). In that study, 1,2-DAG content of spontaneously hypertensive rat hearts during the early stages appeared to be related to the initiation of cardiac hypertrophy in spontaneously hypertensive rats hearts before they developed hypertension (Okumura et al., 1990). Norepinephrine induces diacylglycerol production through its binding to alpha-1 adrenergic receptors, activating PLC and resulting in the generation of DAG (Hussain et al., 2024). In our study, DAG levels were found to be high in hypertensive mice. However, this study did not measure DAG levels in juvenile before inducing hypertension by salt-loading. But, the levels of several DAGs were found to be decreased after hAAT treatment (Figure 4). These studies show that DAGs via acting as a crucial regulator of protein kinase C activity, may have a role in the circulatory alterations associated with hypertension. Moreover, these changes have been shown to start before the onset of hypertension. Overall, the results from this study suggest that hAAT may have therapeutic potential to mitigate hypertension in juvenile mice. Johnsen et al. showed in their study that PKC levels were high in a mouse model of spontaneous hypertensive heart failure (Johnsen et al., 2005). Similarly, Osicka et al. demonstrated that the renal cortex in rat models of hypertension and hyperglycemia, both alone and when combined, exhibit an elevated expression of PKC isoforms (Osicka et al., 2003). In our study, basal levels of PKC activity was found to be higher in salt-loaded 129Sv mice treated with vehicle compared to mice treated with hAAT (Figure 5). Moreover, Wynne et al. showed that diastolic, systolic, and mean arterial pressure of PKC knockout mice were considerably lower compared to the control group (Wynne et al., 2018). PKC has effects beyond decreasing blood pressure. Activation of PKC-δ signaling pathway controls the contraction of the heart muscle and has a crucial function in the development of myocardial ischemia-reperfusion injury and cardiac fibrosis. Studies utilizing pig, rat, and PKC-δ gene deletion mice models demonstrated PKC-δ actively facilitates ischemia/reperfusion injury, and the suppression of PKC-δ can effectively prevent these detrimental effects. Peptide inhibitors administered during reperfusion have a specific effect on protecting the heart. This protection is achieved by decreasing the size of the damaged heart tissue (infarct area) and reducing the levels of troponin T, a protein associated with heart damage. Additionally, these inhibitors improve the recovery of heart function by reducing the activity of caspase-3, an enzyme involved in cell death, and decreasing the apoptosis (Lincoff et al., 2014; Miao et al., 2022). All of these findings indicate that inhibiting PKC could potentially prevent permanent damage to the heart during the restoration of blood flow not only during hypertension. Our data suggest that hAAT may lower PKC activity by reducing DAGs.

One limitation of our study is that we did not compare regional differences in lipids between the cortex and medulla. Another limitation is that we did not measure differences in membrane lipids between male and female mice. We used only male mice in this study since it is established that female sex hormones have a protective role in the development of hypertension (Xue et al., 2007). Third, we did not use other salt-sensitive animal models to corroborate the effects we observed in 129Sv mice. We and other groups have shown 129Sv mice are salt-sensitive and develop hypertension. In contrast, C57Bl6 mice are generally salt resistant to salt loading and are not the preferred model to study the development of hypertension due to dietary sodium. However, our group showed that db/db mice develop hypertension after salt-loading. Future studies will examine whether the lipids we found to be downregulated in 129Sv mice are also downregulated in juvenile salt-loaded hypertensive db/db mice. Another future direction relevant to the scope of this study to investigate whether hAAT administration affects the release and excretion of extracellular vesicles into the urine or the enrichment of bioactive lipids in urinary extracellular vesicles. A previous study showed several hexosylceramides and other classes of lipids are enriched in urinary extracellular vesicles after administration of hAAT in hypertensive diabetic db/db mice (Lugo et al., 2022). Another future direction is to investigate the mechanism by which hAAT is taken up by the thick ascending limb cells expressing NKCC2. Finally, we plan to investigate whether other effector proteins downstream in the DAG-PKC pathway are affected by hAAT treatment. DAG mediated activation of PKC has been shown to result in the activation of protein kinase D (PKD) (Wang, 2006).

**FIGURE 8:**
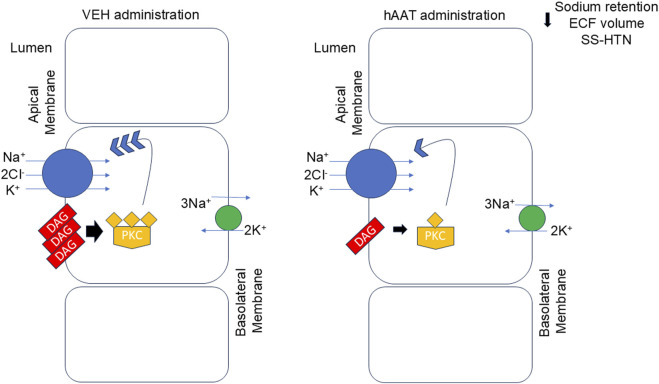
Proposed mechanism for hAAT in mitigating salt-induced hypertension in juvenile mice. In the kidney of salt-induced hypertensive 129Sv mice there is an abundant level of various bioactive diacylglycerols (DAGs) which results in the downstream activation of protein kinase C (PKC). PKC stimulates the insertion and membrane expression of the renal sodium-potassium-chloride co-transporter at the apical plasma membrane. A decrease in surface expression of NKCC2 at the apical plasma membrane is shown to result in a decrease in sodium retention, extracellular fluid (ECF) volume, and hypertension in these animals. hAAT administration reduces the availability of various DAGs at the plasma membrane thus resulting in a decrease in PKC activity. As a result, there is less protein expression of NKCC2 at the apical plasma membrane and less reabsorption and sodium retention in 129Sv mice given hAAT. SS-HTN refers to salt-sensitive hypertension.

Taken together, our descriptive lipidomic dataset identified several DAGs that were attenuated in kidney cortex membrane fractions of salt-loaded hypertensive juvenile 129Sv mice given hAAT compared to vehicle. A reduction in the availability of bioactive DAGs is expected to prevent high levels of PKC activity and subsequently reduce surface expression of renal NKCC2 (Figure 8). Renal NKCC2 plays an important role in regulating total body salt balance and blood pressure. A decrease in its expression and activity is expected to reduce sodium reabsorption in the kidney and mitigate salt-induced hypertension.

## Data Availability

The raw data supporting the conclusions of this article will be made available by the authors, without undue reservation.

## References

[B1] Alli A. Yu L. Holzworth M. Richards J. Cheng K. Y. Lynch I. J. (2019). Direct and indirect inhibition of the circadian clock protein Per1: effects on ENaC and blood pressure. Am. J. Physiol. Ren. Physiol. 316, F807–F813. 10.1152/ajprenal.00408.2018 PMC6580256 30759025

[B2] Alli A. A. Bao H. F. Alli A. A. Aldrugh Y. Song J. Z. Ma H. P. (2012). Phosphatidylinositol phosphate-dependent regulation of Xenopus ENaC by MARCKS protein. Am. J. Physiol. Ren. Physiol. 303, F800–F811. 10.1152/ajprenal.00703.2011 PMC3468524 22791334

[B3] Bao H. F. Thai T. L. Yue Q. Ma H. P. Eaton A. F. Cai H. (2014). ENaC activity is increased in isolated, split-open cortical collecting ducts from protein kinase Cα knockout mice. Am. J. Physiol. Ren. Physiol. 306, F309–F320. 10.1152/ajprenal.00519.2013 PMC3920049 24338818

[B4] Berridge M. J. (1987). Inositol trisphosphate and diacylglycerol: two interacting second messengers. Annu. Rev. Biochem. 56, 159–193. 10.1146/annurev.bi.56.070187.001111 3304132

[B5] Bligh E. G. Dyer W. J. (1959). A rapid method of total lipid extraction and purification. Can. J. Biochem. Physiol. 37, 911–917. 10.1139/o59-099 13671378

[B6] Cai J. Abramovici H. Gee S. H. Topham M. K. (2009). Diacylglycerol kinases as sources of phosphatidic acid. Biochim. Biophys. Acta 1791, 942–948. 10.1016/j.bbalip.2009.02.010 19264149 PMC2731829

[B7] Castaneda-Bueno M. Arroyo J. P. Zhang J. Puthumana J. Yarborough O. 3rd Shibata S. (2017). Phosphorylation by PKC and PKA regulate the kinase activity and downstream signaling of WNK4. Proc. Natl. Acad. Sci. U. S. A. 114, E879–E886. 10.1073/pnas.1620315114 28096417 PMC5293014

[B8] Dathe C. Daigeler A. L. Seifert W. Jankowski V. Mrowka R. Kalis R. (2014). Annexin A2 mediates apical trafficking of renal Na⁺-K⁺-2Cl⁻ cotransporter. J. Biol. Chem. 289, 9983–9997. 10.1074/jbc.M113.540948 24526686 PMC3975042

[B9] Gholam M. F. Liu L. P. Searcy L. A. Denslow N. D. Alli A. A. (2023). Dapagliflozin treatment augments bioactive phosphatidylethanolamine concentrations in kidney cortex membrane fractions of hypertensive diabetic db/db mice and alters the density of lipid rafts in mouse proximal tubule cells. Int. J. Mol. Sci. 24, 1408. 10.3390/ijms24021408 36674924 PMC9865226

[B10] Grimstein C. Choi Y. K. Wasserfall C. H. Satoh M. Atkinson M. A. Brantly M. L. (2011). Alpha-1 antitrypsin protein and gene therapies decrease autoimmunity and delay arthritis development in mouse model. J. Transl. Med. 9, 21. 10.1186/1479-5876-9-21 21345239 PMC3050720

[B11] Haque M. Z. Ortiz P. A. (2019). Superoxide increases surface NKCC2 in the rat thick ascending limbs via PKC. Am. J. Physiol. Ren. Physiol. 317, F99–F106. 10.1152/ajprenal.00232.2018 PMC7139495 31091128

[B12] Hinton T. C. Adams Z. H. Baker R. P. Hope K. A. Paton J. F. R. Hart E. C. (2020). Investigation and treatment of high blood pressure in young people: too much medicine or appropriate risk reduction? Hypertension 75, 16–22. 10.1161/HYPERTENSIONAHA.119.13820 31735086

[B13] Hsu W. F. Kao Y. W. Chen M. Chiang H. C. Chen S. Y. Lu M. C. (2021). A reappraisal of the prevalence of pediatric hypertension through a nationwide database in Taiwan. Sci. Rep. 11, 4475. 10.1038/s41598-021-84001-6 33627680 PMC7904942

[B14] Hussain L. S. Reddy V. Maani C. V. Physiology, Noradrenergic Synapse, StatPearls, Treasure Island (FL) ineligible companies (2024) “Disclosure: vamsi Reddy declares no relevant financial relationships with ineligible companies,” in Disclosure: christopher Maani declares no relevant financial relationships with ineligible companies.

[B15] Johnsen D. D. Kacimi R. Anderson B. E. Thomas T. A. Said S. Gerdes A. M. (2005). Protein kinase C isozymes in hypertension and hypertrophy: insight from SHHF rat hearts. Mol. Cell Biochem. 270, 63–69. 10.1007/s11010-005-3781-x 15792354

[B16] Lincoff A. M. Roe M. Aylward P. Galla J. Rynkiewicz A. Guetta V. (2014). Inhibition of delta-protein kinase C by delcasertib as an adjunct to primary percutaneous coronary intervention for acute anterior ST-segment elevation myocardial infarction: results of the PROTECTION AMI Randomized Controlled Trial. Eur. Heart J. 35, 2516–2523. 10.1093/eurheartj/ehu177 24796339

[B17] Lugo C. I. Liu L. P. Bala N. Morales A. G. Gholam M. F. Abchee J. C. (2022). Human alpha-1 antitrypsin attenuates ENaC and MARCKS and lowers blood pressure in hypertensive diabetic db/db mice. Biomolecules 13, 66. 10.3390/biom13010066 36671451 PMC9856210

[B18] Meng Y. Mynard J. P. Smith K. J. Juonala M. Urbina E. M. Niiranen T. (2024). Pediatric blood pressure and cardiovascular health in adulthood. Curr. Hypertens. Rep. 26, 431–450. 10.1007/s11906-024-01312-5 38878251 PMC11455673

[B19] Miao L. N. Pan D. Shi J. Du J. P. Chen P. F. Gao J. (2022). Role and mechanism of PKC-delta for cardiovascular disease: current status and perspective. Front. Cardiovasc Med. 9, 816369. 10.3389/fcvm.2022.816369 35242825 PMC8885814

[B20] Montgomery D. S. Yu L. Ghazi Z. M. Thai T. L. Al-Khalili O. Ma H. P. (2017). ENaC activity is regulated by calpain-2 proteolysis of MARCKS proteins. Am. J. Physiol. Cell Physiol. 313, C42–C53. 10.1152/ajpcell.00244.2016 28468944 PMC5538800

[B21] Myette R. L. Flynn J. T. (2024). The ongoing impact of obesity on childhood hypertension. Pediatr. Nephrol. 39, 2337–2346. 10.1007/s00467-023-06263-8 38189961

[B22] Ohanian J. Ohanian V. (2001). Lipid second messenger regulation: the role of diacylglycerol kinases and their relevance to hypertension. J. Hum. Hypertens. 15, 93–98. 10.1038/sj.jhh.1001139 11317187

[B23] Okumura K. Kondo J. Shirai Y. Muramatsu M. Yamada Y. Hashimoto H. (1990). 1,2-diacylglycerol content in thoracic aorta of spontaneously hypertensive rats. Hypertension 16, 43–48. 10.1161/01.hyp.16.1.43 2163981

[B24] Osicka T. M. Russo L. M. Qiu M. L. Brammar G. C. Thallas V. Forbes J. M. (2003). Additive effects of hypertension and diabetes on renal cortical expression of PKC-alpha and -epsilon and alpha-tubulin but not PKC-beta 1 and -beta 2. J. Hypertens. 21, 2399–2407. 10.1097/00004872-200312000-00029 14654761

[B35] Pang Z. Lu Y. Zhou G. Hui F. Xu L. Viau C. (2024). MetaboAnalyst 6.0: towards a unified platform for metabolomics data processing, analysis and interpretation. Nucl. Acids. Res. 10.1093/nar/gkae253 PMC11223798 38587201

[B25] Pemberton P. A. Kobayashi D. Wilk B. J. Henstrand J. M. Shapiro S. D. Barr P. J. (2006). Inhaled recombinant alpha 1-antitrypsin ameliorates cigarette smoke-induced emphysema in the mouse. COPD 3, 101–108. 10.1080/15412550600651248 17175673

[B26] Robinson C. H. Hussain J. Jeyakumar N. Smith G. Birken C. S. Dart A. (2024). Long-term cardiovascular outcomes in children and adolescents with hypertension. JAMA Pediatr. 178, 688–698. 10.1001/jamapediatrics.2024.1543 38709137 PMC11217870

[B27] Song C. Yue Q. Moseley A. Al-Khalili O. Wynne B. M. Ma H. (2020). Myristoylated alanine-rich C kinase substrate-like protein-1 regulates epithelial sodium channel activity in renal distal convoluted tubule cells. Am. J. Physiol. Cell Physiol. 319, C589–C604. 10.1152/ajpcell.00218.2020 32639874 PMC7509269

[B28] Song P. Zhang Y. Yu J. Zha M. Zhu Y. Rahimi K. (2019). Global prevalence of hypertension in children: a systematic review and meta-analysis. JAMA Pediatr. 173, 1154–1163. 10.1001/jamapediatrics.2019.3310 31589252 PMC6784751

[B29] Song S. Goudy K. Campbell-Thompson M. Wasserfall C. Scott-Jorgensen M. Wang J. (2004). Recombinant adeno-associated virus-mediated alpha-1 antitrypsin gene therapy prevents type I diabetes in NOD mice. Gene Ther. 11, 181–186. 10.1038/sj.gt.3302156 14712302

[B30] Wang Q. J. (2006). PKD at the crossroads of DAG and PKC signaling. Trends Pharmacol. Sci. 27, 317–323. 10.1016/j.tips.2006.04.003 16678913

[B31] Wynne B. M. McCarthy C. G. Szasz T. Molina P. A. Chapman A. B. Webb R. C. (2018). Protein kinase Cα deletion causes hypotension and decreased vascular contractility. J. Hypertens. 36, 510–519. 10.1097/HJH.0000000000001596 29120956 PMC6287750

[B32] Xue B. Pamidimukkala J. Lubahn D. B. Hay M. (2007). Estrogen receptor-alpha mediates estrogen protection from angiotensin II-induced hypertension in conscious female mice. Am. J. Physiol. Heart Circ. Physiol. 292, H1770–H1776. 10.1152/ajpheart.01011.2005 17142339

[B33] Yamane T. Hachisu R. Yuguchi M. Takeuchi K. Murao S. Yamamoto Y. (2013). Knockdown of legumain inhibits cleavage of annexin A2 in the mouse kidney. Biochem. Biophys. Res. Commun. 430, 482–487. 10.1016/j.bbrc.2012.12.010 23237799

[B34] Yang Y. Min J. Chang L. Chai J. Song Z. Zha S. (2021). Prevalence trends of hypertension among 9-17 aged children and adolescents in Yunnan, 2017-2019: a serial cross-sectional surveillance survey. BMC Public Health 21, 338. 10.1186/s12889-021-10258-1 33579239 PMC7881612

