# Supplementary material for: Alpha 1-antitrypsin mitigates salt-sensitive hypertension in juvenile mice by reducing diacylglycerol concentrations and protein kinase C activity in kidney membranes
Source: Front Mol Biosci. 2025 Jan 20;11:1485506. doi: 10.3389/fmolb.2024.1485506 (PMC11788078; doi:10.3389/fmolb.2024.1485506)
Supplement: Supplementary file 1 [file Table1.docx]

| No | Name | FC | log2(FC) |
| --- | --- | --- | --- |
| 1 | CER(d18:1/18:1)+H | 0.49491 | -1.0148 |
| 2 | DAG(16:0_16:0)+NH4 | 0.35447 | -1.4963 |
| 3 | DAG(16:0_17:0)+NH4 | 0.29433 | -1.7645 |
| 4 | DAG(16:0_18:0)+NH4 | 0.48681 | -1.0386 |
| 5 | DAG(16:0_18:1)+NH4 | 0.41595 | -1.2655 |
| 6 | DAG(16:0_20:0)+NH4 | 0.43985 | -1.1849 |
| 7 | DAG(16:0_20:4)+NH4 | 0.49665 | -1.0097 |
| 8 | DAG(16:0_22:5)+NH4 | 0.38054 | -1.3939 |
| 9 | DAG(16:0_22:6)+NH4 | 0.35821 | -1.4811 |
| 10 | DAG(18:0_18:0)+NH4 | 0.36897 | -1.4384 |
| 11 | DAG(18:0_19:0)+NH4 | 0.48093 | -1.0561 |
| 12 | DAG(18:0_20:3)+NH4 | 0.38307 | -1.3843 |
| 13 | DAG(18:0_20:4)+NH4 | 0.40543 | -1.3025 |
| 14 | DAG(18:0_22:5)+NH4 | 0.43498 | -1.201 |
| 15 | DAG(18:0_22:6)+NH4 | 0.36538 | -1.4525 |
| 16 | DAG(18:1_18:1)+NH4 | 0.32171 | -1.6362 |
| 17 | DAG(18:1_20:4)+NH4 | 0.43487 | -1.2013 |
| 18 | DAG(18:1_22:6)+NH4 | 0.26149 | -1.9352 |
| 19 | DAG(18:2_18:2)+NH4 | 0.26489 | -1.9165 |
| 20 | DAG(18:2_22:6)+NH4 | 0.26829 | -1.8982 |
| 21 | GlcCer(d18:1/16:0)+H | 0.43373 | -1.2051 |
| 22 | GlcCer(d18:1/24:1)+H | 0.3858 | -1.3741 |
| 23 | GlcCer(d18:1/26:0)+H | 0.4058 | -1.3012 |
| 24 | PC(19:0_20:3)+AcO | 2.9671 | 1.5691 |
| 25 | PC(19:0_22:6)+AcO | 0.41269 | -1.2769 |
| 26 | PC(20:1_20:5)+AcO | 2.5782 | 1.3664 |
| 27 | PC(20:2_22:6)+AcO | 0.41482 | -1.2695 |
| 28 | PC(20:3_22:5)+AcO | 2.6502 | 1.4061 |
| 29 | PE(19:0_20:1)-H | 2.6576 | 1.4101 |
| 30 | PE(P-18:1_22:6)+H | 0.47801 | -1.0649 |
| 31 | PG(14:0_14:0)-H | 2.4224 | 1.2764 |
| 32 | PG(14:0_19:0)-H | 2.3529 | 1.2344 |
| 33 | PG(16:0_19:0)-H | 2.9321 | 1.5519 |
| 34 | PG(16:1_19:0)-H | 3.1084 | 1.6362 |
| 35 | PG(17:0_20:1)-H | 3.1471 | 1.654 |
| 36 | PG(17:0_20:2)-H | 3.5893 | 1.8437 |
| 37 | PG(18:0_18:3)-H | 2.4427 | 1.2885 |
| 38 | PG(18:0_19:0)-H | 2.2463 | 1.1675 |
| 39 | PG(18:1_19:0)-H | 2.1804 | 1.1246 |
| 40 | PG(18:2_20:0)-H | 2.4761 | 1.3081 |
| 41 | PG(19:0_20:5)-H | 4.0334 | 2.012 |
| 42 | PG(19:0_22:5)-H | 2.9312 | 1.5515 |
| 43 | PS(14:0_17:0)-H | 3.9397 | 1.9781 |
| 44 | PS(15:0_18:2)-H | 4.2773 | 2.0967 |
| 45 | PS(18:3_20:0)-H | 4.6873 | 2.2288 |
| 46 | TAG(48:1_FA14:0)+NH4 | 0.4179 | -1.2588 |
| 47 | TAG(48:2_FA16:0)+NH4 | 0.4265 | -1.2294 |
| 48 | TAG(48:2_FA18:2)+NH4 | 0.49603 | -1.0115 |
| 49 | TAG(48:3_FA18:2)+NH4 | 0.34105 | -1.552 |
| 50 | TAG(50:2_FA16:0)+NH4 | 0.40487 | -1.3045 |
| 51 | TAG(50:2_FA16:1)+NH4 | 0.37553 | -1.413 |
| 52 | TAG(50:2_FA18:1)+NH4 | 0.3779 | -1.4039 |
| 53 | TAG(50:3_FA14:0)+NH4 | 0.32923 | -1.6028 |
| 54 | TAG(50:3_FA16:0)+NH4 | 0.32985 | -1.6001 |
| 55 | TAG(50:3_FA16:1)+NH4 | 0.36219 | -1.4652 |
| 56 | TAG(50:3_FA18:1)+NH4 | 0.34315 | -1.5431 |
| 57 | TAG(50:3_FA18:2)+NH4 | 0.40564 | -1.3017 |
| 58 | TAG(50:4_FA18:2)+NH4 | 0.29652 | -1.7538 |
| 59 | TAG(52:1_FA16:0)+NH4 | 0.40465 | -1.3052 |
| 60 | TAG(52:1_FA18:0)+NH4 | 0.34626 | -1.5301 |
| 61 | TAG(52:1_FA18:1)+NH4 | 0.41155 | -1.2809 |
| 62 | TAG(52:2_FA16:0)+NH4 | 0.38652 | -1.3714 |
| 63 | TAG(52:2_FA18:0)+NH4 | 0.40368 | -1.3087 |
| 64 | TAG(52:2_FA18:1)+NH4 | 0.33905 | -1.5604 |
| 65 | TAG(52:3_FA16:0)+NH4 | 0.33932 | -1.5593 |
| 66 | TAG(52:3_FA18:0)+NH4 | 0.37288 | -1.4232 |
| 67 | TAG(52:3_FA18:1)+NH4 | 0.32189 | -1.6354 |
| 68 | TAG(52:3_FA18:2)+NH4 | 0.32883 | -1.6046 |
| 69 | TAG(52:3_FA18:3)+NH4 | 0.33677 | -1.5702 |
| 70 | TAG(52:4_FA16:0)+NH4 | 0.34346 | -1.5418 |
| 71 | TAG(52:4_FA16:1)+NH4 | 0.32403 | -1.6258 |
| 72 | TAG(52:4_FA18:2)+NH4 | 0.3609 | -1.4703 |
| 73 | TAG(52:4_FA18:3)+NH4 | 0.35754 | -1.4838 |
| 74 | TAG(52:5_FA16:0)+NH4 | 0.41428 | -1.2713 |
| 75 | TAG(52:5_FA16:1)+NH4 | 0.27549 | -1.8599 |
| 76 | TAG(52:5_FA18:2)+NH4 | 0.31273 | -1.677 |
| 77 | TAG(52:5_FA18:3)+NH4 | 0.30306 | -1.7223 |
| 78 | TAG(53:0_FA16:0)+NH4 | 0.48034 | -1.0579 |
| 79 | TAG(53:3_FA18:2)+NH4 | 0.31459 | -1.6685 |
| 80 | TAG(54:2_FA20:1)+NH4 | 0.29038 | -1.784 |
| 81 | TAG(54:3_FA16:0)+NH4 | 0.43107 | -1.214 |
| 82 | TAG(54:3_FA18:1)+NH4 | 0.36551 | -1.452 |
| 83 | TAG(54:3_FA18:2)+NH4 | 0.34209 | -1.5475 |
| 84 | TAG(54:4_FA18:1)+NH4 | 0.29315 | -1.7703 |
| 85 | TAG(54:4_FA18:2)+NH4 | 0.35005 | -1.5144 |
| 86 | TAG(54:5_FA18:1)+NH4 | 0.28226 | -1.8249 |
| 87 | TAG(54:5_FA18:2)+NH4 | 0.24402 | -2.035 |
| 88 | TAG(54:5_FA18:3)+NH4 | 0.2904 | -1.7839 |
| 89 | TAG(54:6_FA18:1)+NH4 | 0.27611 | -1.8567 |
| 90 | TAG(54:6_FA18:2)+NH4 | 0.3677 | -1.4434 |
| 91 | TAG(54:6_FA18:3)+NH4 | 0.28344 | -1.8189 |
| 92 | TAG(54:7_FA18:2)+NH4 | 0.35879 | -1.4788 |
| 93 | TAG(54:7_FA18:3)+NH4 | 0.31265 | -1.6774 |
| 94 | TAG(56:3_FA20:1)+NH4 | 0.49574 | -1.0123 |
| 95 | TAG(56:4_FA18:1)+NH4 | 0.38698 | -1.3697 |
| 96 | TAG(56:6_FA18:1)+NH4 | 0.47412 | -1.0767 |

**Supplementary Table 1.** Lipidomic dataset of lipids in kidney cortex membrane fractions of 129Sv mice treated with hAAT or vehicle. CER represents ceramides, DAG represents diacylglycerols, GlcCer represents glucosylceramides, PC represents phosphatidylcholine, PE represents phosphatidylethanolamine. PEP represents phosphatidylethanolamine plasmogen, PG represents phosphatidylglycerols, PS represents phosphatidylserine, TAG represents triacylglyerols. FC refers to fold change.
