# Supplementary figures and images for: Alpha 1-antitrypsin mitigates salt-sensitive hypertension in juvenile mice by reducing diacylglycerol concentrations and protein kinase C activity in kidney membranes
Source: Front Mol Biosci. 2025 Jan 20;11:1485506. doi: 10.3389/fmolb.2024.1485506 (PMC11788078; doi:10.3389/fmolb.2024.1485506)

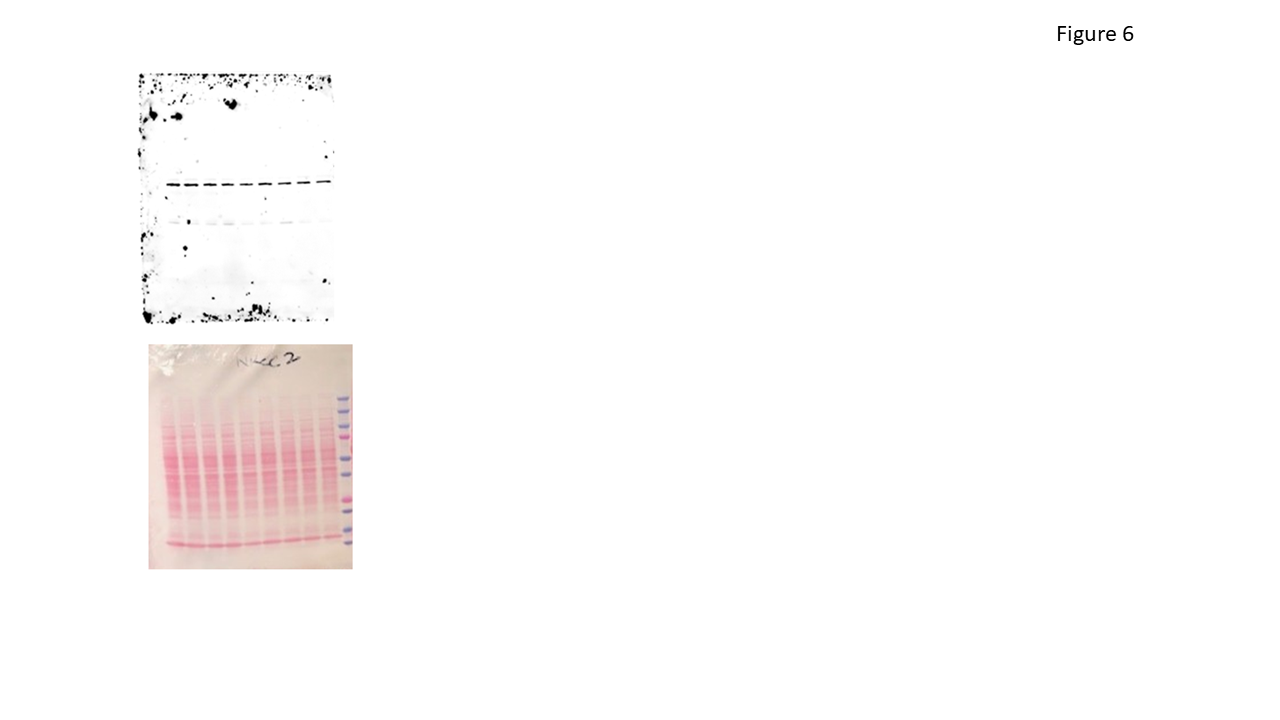

Supplement: Supplementary file 2 [file Image1.tif]
